# Chloroquine and Hydroxychloroquine in the Treatment of Dry Eye Disease

**DOI:** 10.3390/diseases11020085

**Published:** 2023-06-12

**Authors:** Julia Prinz, Nicola Maffulli, Matthias Fuest, Peter Walter, Frank Hildebrand, Filippo Migliorini

**Affiliations:** 1 RWTH University Hospital of Aachen, Pauwelsstraße 30, 52074 Aachen, Germanymigliorini.md@gmail.com (F.M.); 2 Department of Medicine, Surgery and Dentistry, University of Salerno, 84081 Fisciano, Italy; 3 Barts and The London School of Medicine and Dentistry, Mile End Hospital, Queen Mary University of London, 275 Bancroft Road, London E1 4DG, UK; 4 School of Pharmacy and Bioengineering, Keele University Faculty of Medicine, Thornburrow Drive, Stoke on Trent ST4 7QB, UK; 5 Department of Orthopaedics and Trauma Surgery, Academic Hospital of Bolzano (SABES-ASDAA), 39100 Bolzano, Italy

**Keywords:** chloroquine, hydroxychloroquine, dry eye disease, xerophthalmus, keratoconjunctivitis sicca

## Abstract

The role of chloroquine (CQ) and hydroxychloroquine (HCQ) in the management of dry eye disease is still unclear. This systematic review and meta-analysis investigates the efficacy and feasibility of CQ and HCQ in patients with dry eye disease. In February 2023, PubMed, Embase, Google Scholar, and Web of Science were accessed. Data from 462 patients (mean age 54.4 ± 2.8 years) were collected. Compared to baseline, the tear breakup time (*p* < 0.0001) and Schirmer I test (*p* < 0.0001) were significantly increased, and the Ocular Surface Disease Index (OSDI, *p* < 0.0001) and corneal staining (*p* < 0.0001) were significantly decreased at the last follow-up in the CQ/HCQ group. At the last follow-up, the OSDI was significantly lower in the CQ/HCQ group compared to the control group (*p* < 0.0001). Corneal staining was significantly greater in the control group compared to the CQ/HCQ group (*p* < 0.0001). The Schirmer I test showed no significant difference between the groups (*p* = 0.2). Altogether, CQ and HCQ improved the symptoms and signs of dry eye disease.

## 1. Introduction

Dry eye disease is a multifactorial ocular condition [1]. Its prevalence is estimated to be as high as 50% in adults [2]. The aqueous-deficient dry eye disease subtype includes Sjögren’s-syndrome-related dry eye disease with associated systemic diseases, such as rheumatoid arthritis, or systemic sclerosis, and non-Sjögren’s-syndrome-related dry eye disease (Table 1) [3]. The evaporative dry eye disease subtype comprises Meibomian gland diseases, ocular-surface-related evaporative dry eye disease forms, or disorders of the lid aperture [3]. In some dry eye disease patients, both subtypes can coexist [3].

Patients with dry eye disease might suffer from photophobia, pain, or impaired vision [4,5]. Different autoimmune disorders, including thyroid diseases, environmental conditions, including contact lens wear or cigarette smoking, vitamin A deficiency, diabetes mellitus, and hormonal disbalances can be implicated in dry eye disease [5,6,7,8].

The condition affects the tear film and leads to damage of the ocular surface [9]. A desiccating stress followed by a vicious cycle of ocular surface inflammation plays an important role in the pathogenesis of dry eye disease [3,5,10]. Increased expression of inflammatory cytokines and chemokines in the epithelium of the ocular surface [11] and in the tear fluid [12] have been found in eyes suffering from dry eye disease. Damage to the lacrimal gland (e.g., acute due to radiation exposure or chronic in patients with autoimmune diseases, such as Sjögren’s syndrome) may lead to an infiltration of the lacrimal gland by lymphocytes [3,13,14].

The Schirmer I test [15], tear breakup time test [16], and corneal staining [17] are used to diagnose dry eye disease. Additionally, the patient’s subjective burden is evaluated by a number of questionnaires, including the Ocular Surface Disease Index (OSDI) [18].

Currently, artificial tears are the mainstay of treatment for dry eye disease [19]. However, artificial tears only yield symptomatic improvement and do not treat the underlying pathomechanism of the disease, involving inflammation of the ocular surface [19]. The efficacy of various topical anti-inflammatory agents, such as corticosteroids, lifitegrast, and cyclosporine in the treatment of dry eye disease compared against placebo has been shown in previous studies [20,21,22,23]. Additionally, the efficacy and feasibility of a range of complementary medicines in patients with dry eye disease has been demonstrated, including herbal and natural products [24,25], honey [24,26], or acupuncture [24,27]. However, there is a need for further treatment options targeting different aspects of the disease [28].

Chloroquine (CQ) and hydroxychloroquine (HCQ), a metabolite of CQ with lower toxicity, are well-established anti-inflammatory drugs [29]. CQ and HCQ are commonly used in the management of several conditions which are related to elevated levels of tumor necrosis factor α (TNF-α) [30], including malaria [30], discoid lupus arthritis [31], or rheumatoid arthritis [32]. In HCQ, an N-ethyl substituent of CQ is ß-hydroxylated [33]. CQ and HCQ show similar pharmacokinetic properties [33,34]. Their efficacy is attributed to a high ability to penetrate tissues as well as a high oral bioavailability [33]. CQ and HCQ are known to have anti-inflammatory, immunosuppressive, and immunomodulatory effects [34,35]. The way of action of CQ and HCQ includes an inhibition of endosomal toll-like receptor signaling, ultimately decreasing cytokine production [36,37]. Further mechanisms involve the inhibition of mitogen-activated protein kinase (MAPK) signaling and a reduction of matrix metalloproteinase 9 activity [38,39]. Recently, the efficacy of CQ and HCQ in the treatment of dry eye disease has been investigated in the clinical setting [19,40,41,42,43,44].

Currently, a detailed literature review of the efficacy of CQ and HCQ in dry eye disease is still warranted. Therefore, the present systematic review and meta-analysis aims to analyze the efficacy of CQ and HCQ in patients with dry eye disease. The primary outcome of interest was to examine whether CQ and HCQ improve the signs and symptoms of dry eye disease from baseline to the last follow-up. The secondary outcome of interest was to compare CQ and HCQ with placebo or artificial tears.

**Table 1:** Aqueous-deficient and evaporative dry eye disease subtypes (according to Bron et al., TFOS DEWS II pathophysiology report [3]).

Aqueous-Deficient Dry Eye Disease	Evaporative Dry Eye Disease
Sjögren’s-syndrome-related dry eye disease [3,45]	Meibomian Gland Dysfunction [3]
Non-Sjögren’s-syndrome-related dry eye disease, including inflammatory lacrimal gland infiltration, or lacrimal gland obstruction [3,45]	Ocular surface related evaporative dry eye disease [3]
Other conditions, including diabetes mellitus [3]	Disorders of the lid aperture [3]

## 2. Materials and Methods

### 2.1. Eligibility Criteria

All randomized controlled trials analyzing the efficacy of CQ and HCQ for dry eye disease were considered. Articles in English, German, Italian, French, Dutch, and Spanish were accessed. Only level I evidence studies (Oxford Centre of Evidence-Based Medicine) [46] were examined. Reviews, editorials, opinions, and letters were not accessed. Additionally, computational, biomechanics, animal, in vitro, and cadaveric studies were not included.

### 2.2. Search Strategy

This study was performed according to the 2020 PRISMA statement (Preferred Reporting Items for Systematic Reviews and Meta-Analyses) [47]. The PICO algorithm was identified as follows:

- P (Population): patients with dry eye disease;
- I (Intervention): treatment with CQ or HCQ;
- C (Comparison): efficacy at the last follow-up, comparison with placebo or artificial tears (control group);
- O (Outcomes): Tear breakup time; Schirmer I test, corneal staining, Ocular Surface Disease Index.

In February 2023, PubMed, Embase, Google Scholar, and Web of Science were searched without time constraints. We used the following keywords: dry eye disease xerophthalmus, xerophthalmia, keratoconjunctivitis sicca, aqueous deficient dry eye disease, evaporative dry eye disease, TBUT (tear breakup time), tear breakup time, SIT (Schirmer I test), Schirmer I test, chloroquine, hydroxychloroquine, OSDI (Ocular Surface Disease Index), Ocular Surface Disease Index, corneal staining.

### 2.3. Selection and Data Collection

The abstracts of the suitable titles were accessed and the full texts of the matching abstracts were retrieved. At baseline and at the last follow-up, study generalities (including author, year of publication, the number of patients, the percentage of female patients included in the study, and the mean age of all patients), tear breakup time test (TBUT) [16], Ocular Surface Disease Index (OSDI) [48], and Schirmer I test [15] were extracted.

### 2.4. Assessment of the Study Risk of Bias

The risk of bias tool of the Review Manager software (The Nordic Cochrane Collaboration, Copenhagen, Denmark) was employed to estimate the between-studies risk of bias. The selection bias, performance bias, detection bias, attrition bias, reporting bias, and other sources of bias were evaluated by an author independently (J. P.).

### 2.5. Synthesis Methods

The statistical analysis of this study was carried out by the senior author (F. M.). The IBM SPSS software version 25 was used to evaluate any changes from baseline to the last follow-up. The mean difference, standard error, and *T*-test were assessed. A meta-analysis was performed with the Review Manager software (The Nordic Cochrane Collaboration, Copenhagen) version 5.3. Data were evaluated using the inverse variance and mean difference effect measures. The comparisons in this study were conducted with a fixed model effect as set-up. Heterogeneity was analyzed using the Higgins-I^2^ test. If the I^2^ test score was >50%, we adopted a random model effect. We used 95% confidence intervals (CI) for all 95% analyses. If *p* < 0.05, the values or comparisons were statistically significant. Forest plots were conducted for all comparisons.

## 3. Results

### 3.1. Study Selection

**Figure 1:**
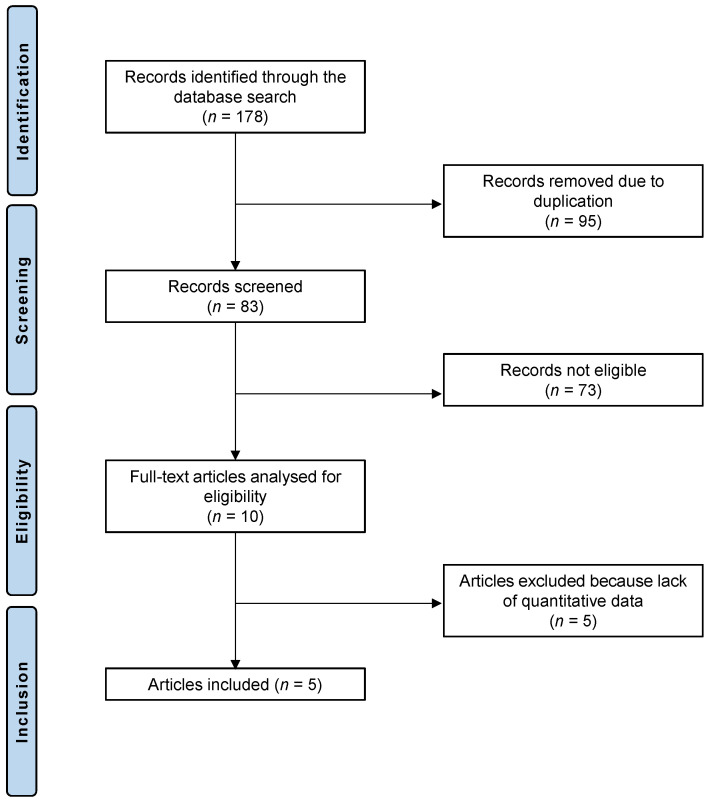
PRISMA diagram of the literature research.

The literature search led to 178 randomized controlled trials that analyzed the feasibility and efficacy of HCQ or CQ in patients with dry eye disease. Of these 178 articles, 95 showed redundancy and were therefore not considered. We further excluded 73 articles for not meeting the eligibility criteria: 33 studies were not suitable because of the type of study, 28 studies did not focus on the topic, and 6 studies were not eligible due to language incompatibilities, 6 further studies combined HCQ or CQ therapy with other therapies or techniques and were excluded from the analysis. Five further studies were excluded because no quantitative data could be accessed for the outcomes of interest. Five randomized controlled trials were considered for the final analysis. The PRISMA diagram of the literature research is depicted in Figure 1.

### 3.2. Risk of Bias Evaluation

**Figure 2:**
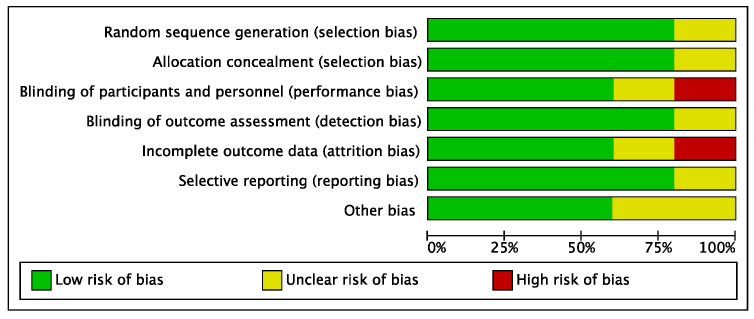
Risk of bias diagram including selection, performance, detection, attrition, reporting, and other biases. Low risk of bias is shown in green, unclear risk of bias in yellow, and high risk of bias in red.

The risk of performance and attrition biases of the included studies can be considered low to moderate. The risks of selection, detection, and reporting biases were low. Overall, the risk of bias in the present systematic review and meta-analysis was low to moderate (Figure 2).

### 3.3. Study Characteristics and Results of Studies

Data from 462 patients were collected. The mean follow-up was 20.8 ± 16.8 weeks. The mean age of the patients was 54.4 ± 2.8 years. The generalities of the studies and patient baseline data are shown in Table 2.

### 3.4. Efficacy of CQ and HCQ

The tear breakup time was significantly increased at the last follow-up compared to baseline (*p* < 0.0001, Table 3). The Schirmer I test was significantly increased (*p* < 0.0001), and corneal staining was significantly reduced at the last follow-up compared to baseline (*p* < 0.0001). The OSDI score was significantly reduced at the last follow-up compared to baseline (*p* < 0.0001). The mean values and standard deviations at baseline and at the last follow-up are displayed in Table 3.

### 3.5. CQ and HCQ Compared to Other Treatments

**Figure 3:**
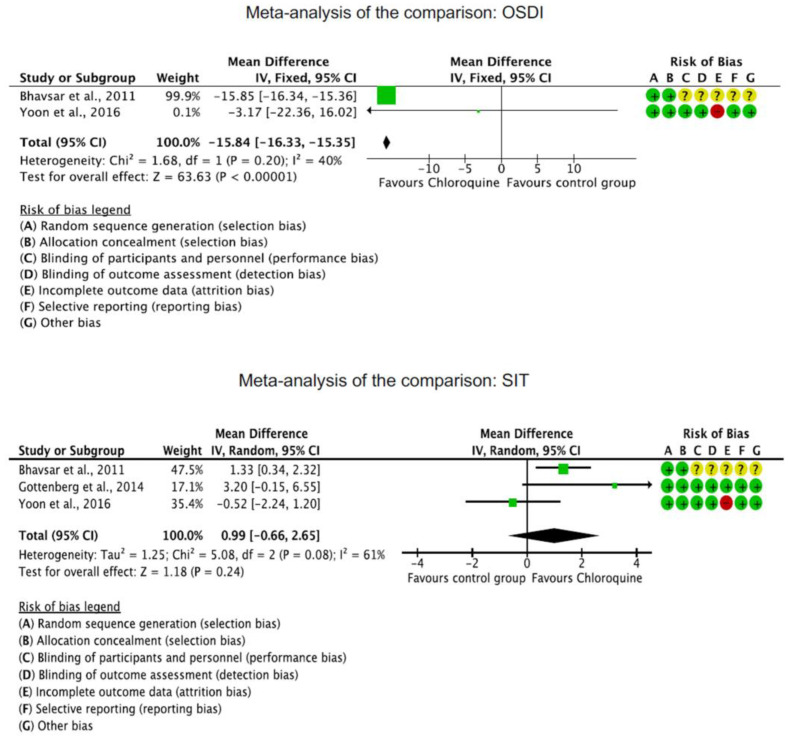

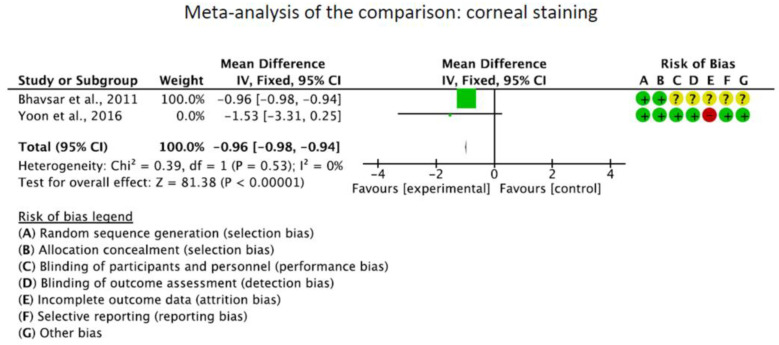
Results of the meta-analyses [19,40,41].

The OSDI score was significantly reduced in the CQ/HCQ group compared to the control group (MD −15.84; 95%CI −16.33 to −15.35; *p* < 0.0001). The Schirmer I test showed no significant difference between the CQ/HCQ and the control group (*p* = 0.2). Corneal staining was greater in the control group (MD −0.96; 95%CI −0.99 to 0.93; *p* < 0.0001). These results are shown in greater detail in Figure 3.

## 4. Discussion

In the present meta-analysis and systematic review including five randomized controlled trials, the Schirmer I test and the tear breakup time were significantly increased at the last follow-up compared to baseline in the CQ/HCQ group. At the last follow-up, the OSDI score and corneal staining were significantly lower compared to baseline in the CQ/HCQ group. The OSDI score and corneal staining were lower in the CQ/HCQ group compared to the control group at the last follow-up, whereas Schirmer I test values were similar. Altogether, treatment with CQ and HCQ might be a feasible and effective treatment strategy to reduce the signs and symptoms of dry eye disease. However, these results must be interpreted in the light of the limitations of the present study, especially the small number of included studies and methodological approaches.

In 2017, a systematic review and meta-analysis by Wang et al. revealed no significant difference between HCQ and a placebo in the management of dry eye disease in patients with Sjögren’s syndrome [49]. This meta-analysis included only patients with Sjögren’s syndrome. In contrast, the present study collected data from patients with different subtypes of dry eye disease. Four trials with a total of 215 patients were included in the study by Wang et al., among them the studies by Yoon et al. [40] and Gottenberg et al. [41]. In addition, a retrospective and a cross-over study were considered [49]. The authors reported a slightly higher effect of HCQ in dry eye disease compared to placebo [49]. However, no significant effect occurred. In addition, gastrointestinal adverse effects were associated with HCQ therapy [49]. As only two randomized clinical trials were included in this study by Wang et al. [49], the present systematic review and meta-analysis aims to analyze the efficacy and feasibility of CQ and HCQ in patients with dry eye disease according to the randomized clinical trials presented in the current literature. In addition, our study included both patients with Sjögren’s-syndrome-related dry eye disease and non-Sjögren’s-syndrome-related dry eye disease.

Yoon et al. analyzed the 12-week outcomes of treatment with oral HCQ in 26 patients with Sjögren’s-syndrome-related dry eye disease in a double-blind randomized controlled trial. The patients were allocated to either treatment with oral HCQ (300 mg/daily) or oral placebo. At the last follow-up (16 weeks after drug discontinuance), the Schirmer I test and tear breakup time values did not change significantly after treatment with HCQ and showed no difference between the HCQ and the placebo group, suggesting no relevant effect of HCQ on tear production. The subjective symptoms evaluated by the OSDI improved post-treatment with HCQ. No significant difference occurred between the HCQ and the placebo group [40]. However, the sample size was limited in the study by Yoon et al. [40].

Bhavsar et al. analyzed the efficacy of CQ phosphate eye drops compared to sodium carboxymethyl cellulose for the treatment of dry eye disease in 170 patients with non-Sjögren’s-syndrome-related dry eye disease. The authors found a significant increase in Schirmer I test values at 2, 3, and 4 weeks compared to baseline in the CQ groups [19]. In patients receiving sodium carboxymethyl cellulose eye drops, a significant increase in Schirmer I test score was witnessed at the three-week follow-up only [19]. The Schirmer I test values increased by 20% in the CQ group and by 9% in the sodium carboxymethyl cellulose group [19]. In addition, Bhavsar et al. reported a significant decrease in the OSDI scores at all follow-ups in both the CQ and the sodium carboxymethyl cellulose groups [19]. Moreover, no relevant side effects due to CQ were reported, which suggests that CQ eye drops might be an effective and feasible treatment option in patients with dry eye disease [19]. Topical CQ therapy might offer important advantages as compared to systemic therapy, possibly avoiding long-term systemic complications [19]. The authors argue that adverse effects and the toxicity of CQ are attributed to a high cumulative systemic dose which might not be achieved by twice daily topical application with eye drops [19]. Tyagi et al. compared the efficacy of CQ phosphate 0.03% eye drops with sodium carboxymethyl cellulose 1% eye drops in a prospective randomized controlled trial including 100 patients with non-Sjögren’s-syndrome-related dry eye disease [44]. Both CQ phosphate and carboxymethyl cellulose were effective in treating dry eye disease, with a faster onset of efficacy of CQ phosphate therapy concerning Schirmer I test and ocular surface staining [44].

Gottenberg et al. analyzed the efficacy of HCQ (400 mg daily) compared to a placebo in patients with Sjögren’s syndrome in the JOQUER randomized clinical trial. Forty-eight patients completed the 48-week follow-up in the placebo group while 44 patients completed the follow-up in the HCQ group. As the primary endpoint, the authors defined the proportion of patients with at least 30% reduction of ocular dryness as estimated by patient assessment. At 24 weeks, the use of HCQ did not improve dry eye disease symptoms compared to placebo. Gottenberg et al. concluded that previous studies possibly overestimated the efficacy of HCQ in patients with Sjögren’s syndrome [41]. In a randomized controlled trial by Bodewes et al., 77 patients, who were previously enrolled in the JOQUER study by Gottenberg et al., were included [41]. The authors reported that treatment for 24 weeks with HCQ reduced type I interferon scores but failed to improve the clinical response [43].

Some previous studies demonstrated that HCQ may alleviate the signs and symptoms of dry eye disease in patients with Sjögren’s syndrome [50], whereas others reported no beneficial effect of HCQ in dry eye disease [42,51]. In the present study, only two studies included OSDI [19,40], two investigated the corneal staining [40,41], two reported on tear breakup time [40,44], and three studies reported on Schirmer I test [19,40,41]. Three studies reported adverse events of CQ/HCQ. Gottenberg et al. found two serious adverse events in the HCQ group including 56 patients (urinary lithiasis, breast cancer), and three in the placebo group including 64 patients (surgery for meningioma, lipothymia, Eppstein–Barr Virus (EBV) and cytomegalovirus (CMV) pneumonia) [41]. However, in the study by Gottenberg et al., the occurrence of serious adverse events did not differ significantly between the HCQ (3.6%) and the placebo group (4.7%), suggesting HCQ to be a safe treatment option. Bhavsar et al. reported minor adverse events, such as conjunctival hyperemia and burning eye without significant differences between the CQ and the control group [19]. Bhavasar et al. analyzed the outcomes of CQ administered topically as eye drops for 21 days. Thus, short-term topical administration of CQ did not lead to serious adverse events in this randomized controlled trial [19]. In the study by Yoon et al., three patients in the HCQ group suffered dyspepsia and one patient developed subretinal hemorrhage from occult myopic choroidal neovascularization, which was not considered as a HCQ-related complication. No serious adverse events occurred [40]. Additionally, possible long-term complications were not considered in the included studies. Treatment with CQ and HCQ may lead to different adverse effects, such as headache or gastrointestinal symptoms [33]. In the eye, they can adversely affect the retina, cornea, and ciliary body [52]. Several adverse effects, including retinopathy and QT-interval prolongation seen in electrocardiogram, might occur during long-term CQ and HCQ therapy [53]. Therefore, CQ and HCQ therapy should not be administered without regular ophthalmological and electrocardiogram investigations [54].

The present study has several limitations. Firstly, the HCQ and CQ administration protocols evaluated differed from one study to the other, and included oral HCQ [40,41,43] and topical CQ application [19,44] in different dosages. The assumption that topical short-term administration of CQ eye drops might not lead to serious adverse events in contrast to systemic administration should be addressed by future larger cohort randomized controlled trials.

The variability in treatment protocols resulted in high heterogeneity in the included articles. Given the limited quantitative data available in the literature for inclusion in the present systematic review and meta-analysis, it was not possible to analyze different application modes or dosages of CQ and HCQ separately. Additionally, the control groups of the included studies were heterogeneous, including artificial tears and placebo: In the studies by Yoon et al. [40], Gottenberg et al. [41], and Bodewes et al. [43], oral HCQ was compared against a placebo, whereas in the studies by Bhavsar et al. [19] and Tyagi et al. [44], CQ eye drops were compared against artificial tears. Given the limited quantitative data available, no subgroup analysis for different control groups was performed.

Moreover, it is questionable whether the outcomes of interest in the present study, especially corneal staining, were collected in an identical fashion in all included studies, given the different investigators and grading scales. Another limitation of the present systematic review and meta-analysis is the small number of randomized controlled trials which were eligible for inclusion. However, this represents the lack of evidence in the literature. Randomized controlled studies are warranted to further investigate the role of CQ and HCQ in patients with evaporative and aqueous-deficient dry eye disease. Furthermore, no subgroup analysis was performed between patients with Sjögren’s-syndrome-related dry eye disease and non-Sjögren’s-syndrome-related dry eye disease because of the small sample sizes. Therefore, results from the present systematic review and meta-analysis must be interpreted within the aforementioned limitations.

## 5. Conclusions

According to the main findings of the present systematic review and meta-analysis, treatment with CQ and HCQ might be an effective and feasible treatment strategy to reduce symptoms and signs of dry eye disease. CQ and HCQ led to a significant increase in the tear breakup time and Schirmer I test and to a significant decrease in the OSDI score and corneal staining at the last follow-up compared to baseline. CQ and HCQ resulted in a significantly lower OSDI score and corneal staining than the control group. Future high-quality long-term studies should focus on possible long-term side effects of systemic and topical administration of CQ or HCQ. Additionally, future studies should analyze the efficacy of CQ and HCQ in different subtypes of dry eye disease, such as Sjögren’s syndrome and non-Sjögren’s-syndrome-related dry eye disease.

## Figures and Tables

**Table 2:** Generalities and patient baseline of the included studies. CQ: chloroquine, HCQ: hydroxychloroquine.

Author, Year	Journal	Follow-Up (Weeks)	Patients (*n*)	Treatment	Dose	Mean Age	Women (*%*)
Bhavsar et al., 2011 [19]	*Int. J. Biomed. Adv. Res.*	4	82	CQ (eye drops)	0.03%	53.4	71
			85	Control group (artificial tears)		53.4	71
Bodewes et al., 2020 [43]	*Rheumatology (Oxford)*	24	37	HCQ (oral)	400 mg daily		
			40	Control group (placebo)			
Gottenberg et al., 2014 [41]	*J. Am. Med. Ass.*	48	44	HCQ (oral)	400 mg daily	56.3	89
			48	Control group (placebo)		55.6	94
Tyagi et al., 2021 [44]	*Ind. J. Clin. Exp. Ophthalm*	12	50	CQ (eye drops)	0.03%	51.0	
			50	Control group (artificial tears)		51.0	
Yoon et al., 2016 [40]	*J. Korean. Med. Sci.*	12	11	HCQ (oral)	300 mg daily	59.4	100
			15	Control group (placebo)		55.0	100

**Table 3:** Values of the tear breakup time (s), OSDI (Ocular Surface Disease Index, points), Schirmer I test (mm), and corneal staining (points) in the chloroquine/hydroxychloroquine group. Data were compared from baseline to the last follow-up (FU). (MD: mean difference; SE: standard error; 95% CI: 95% confidence interval). The tear breakup time (*p* < 0.0001) and Schirmer I test (*p* < 0.0001) were significantly increased at the last follow-up compared to baseline. At the last follow-up, the OSDI score (*p* < 0.0001) and corneal staining (*p* < 0.0001) were significantly lower compared to baseline.

Endpoint	Baseline	Last FU	MD	SE	95% CI	T Value	*p*
Tear breakup time	4.6 ± 1.6	6.9 ± 6.6	2.3	0.453	1.40 to 3.19	5.077	<0.0001
OSDI	57.8 ± 5.2	22.2 ± 7.8	−35.6	0.621	−36.81 to −34.38	−57.357	<0.0001
Schirmer I test	7.4 ± 3.9	10.0 ± 5.1	2.6	0.424	1.76 to 3.43	6.128	<0.0001
Corneal staining	3.2 ± 0.02	1.5 ± 1.5	−1.7	0.1	−1.89 to −1.50	−16.96	<0.0001

## Data Availability

Not applicable.
